# Supplementary material for: Safety and Efficacy of Intravenous Alteplase before Endovascular Thrombectomy: A Pooled Analysis with Focus on the Elderly
Source: J Clin Med. 2022 Jun 26;11(13):3681. doi: 10.3390/jcm11133681 (PMC9267603; doi:10.3390/jcm11133681)
Supplement: Supplementary file 1 [file jcm-11-03681-s001.zip › jcm-1753117-supplementary.pdf]

Supplemental Table S1 Baseline characteristics by age group

	<80 years N=208	≥80 years N=101	P value
Age (SD)	63.38 (12.3)	85.79 (4.6)	-
Gender male (%)	117 (56)	27 (27)	**<0.001**
Hypertension (%)	127 (61)	82 (82)	**<0.001**
Diabetes (%)	63 (30)	31 (30)	0.944
Hyperlipidemia (%)	96 (46)	47 (46)	0.952
Smoking (%)	72 (34)	13 (13)	**<0.001**
Atrial fibrillation (%)	67 (31)	50 (49)	**0.03**
Ischemic heart disease (%)	65 (31)	37 (36)	0.347
Valve disease (%)	14 (7)	4 (4)	0.211
Congestive heart failure (%)	11 (5)	10 (10)	0.224
Chronic renal failure (%)	10 (5)	9 (9)	0.149
Prior stroke (%)	30 (15)	13 (13)	0.631
Malignancy (%)	25 (12)	7 (7)	0.169
Statins (%)	45 (22)	30 (30)	0.073
Antiplatelets (%)	53 (26)	34 (34)	0.113
**Vessel lesion (%)**			
ICA	53 (25)	27 (27)	
M1 MCA	110 (52)	57 (57)	
M2 MCA	27 (13)	15 (15)	0.457
Basilar	12 (6)	5 (2)	
ACA	4 (2)	0 (0)	
PCA	0 (0)	0 (0)	
Tandem lesion	33 (17)	14 (14)	0.649
**TOAST (%)**			
Cardioembolism	115 (55)	68 (68)	
Large- artery atherosclerosis	49 (23)	16 (16)	0.053
Other determined etiology	10 (5)	1 (1)	
Undetermined	32 (15)	14 (14)	
Collaterals (IQR)	2 (1-4)	3 (1-4)	0.771
Symptom to door (SD)	86.1 (61.5)	96.5 (63.2)	0.167
Door to imaging (SD)	29.9 (41.3)	28.5 (40.8)	0.779
Symptom to groin puncture (SD)	253.1 (197.7)	225.6 (119.4)	0.202
Door to groin puncture (SD)	167.8 (180.9)	128.8 (99.7)	**0.048**
tPA (%)	96 (46)	51 (51)	0.474
Number of passes (IQR)	1 (1-3)	1 (1-3)	0.406
TICI 2b- 3 (%)	176 (85)	78 (81)	0.406
First pass (%)	80 (38)	40 (40)	0.653
Stent (%)	33 (16)	7 (7)	**0.027**
ICH (%)	39 (19)	15 (15)	0.466
PH2 (%)	11 (5)	3 (3)	0.399
Symptomatic ICH (%)	6 (3)	2 (2)	0.650
NIHSS on admission (IQR)	15 (10-20)	19 (14-22)	**<0.001**
Discharge NIHSS (IQR)	4 (1-8)	5 (1-14)	0.079
mRS baseline (IQR)	0 (0-0)	1 (0-3)	**<0.001**
mRS-90 (IQR)	2 (1-4)	4 (3-6)	**<0.001**
Favorable outcome (%)	106 (53)	28 (28)	**<0.001**
Mortality (%)	19 (9)	23 (23)	**0.001**

Values represent number of patients unless otherwise stated. Bold numbers signify statistically significant p values. SD = standard deviation, IQR = interquartile range, ICA = internal carotid artery, MCA = middle cerebral artery, ACA = anterior cerebral artery, PCA = posterior cerebral artery, HT = hemorrhagic transformation, PH = parenchymal hematoma. MRS = modified Rankin Scale.

Supplemental Table S2 Patients below 80 years of age

	Direct-EVT N=112	IVT-EVT N=96	P value
Age (SD)	62.5 (13.2)	64.3 (11.1)	0.291
Sex male (%)	60 (53.6)	57 (59.4)	0.4
Hypertension (%)	69 (61.6)	58 (60.4)	0.861
Diabetes (%)	31 (27.7)	32 (33.3)	0.376
Hyperlipidemia (%)	52 (46.4)	43 (44.8)	0.813
Smoking (%)	34 (30.4)	38 (39.6)	0.163
Atrial fibrillation (%)	34 (30.4)	33 (34.4)	0.536
Ischemic heart disease (%)	30 (26.8)	35 (36.5)	0.134
Valve disease (%)	6 (4.5)	9 (6.3)	0.793
Congestive heart failure (%)	5 (4.5)	6 (7.8)	0.312
Chronic renal failure (%)	3 (2.7)	7 (7.3)	0.121
Prior stroke (%)	13 (11.6)	17 (17.7)	0.212
Malignancy (%)	16 (14.3)	9 (9.4)	0.278
Statins (%)	26 (24.4)	17 (19.8)	0.473
Antiplatelets (%)	28 (25.5)	25 (26.3)	0.888
**Vessel lesion (%)**			
ICA	30 (26.8)	22 (22.9)	0.411
M1 MCA	59 (52.7)	51 (53.1)	
M2 MCA	13 (11.6)	14 (14.6)	
Basilar	5 (4.5)	7 (7.3)	
ACA	3 (2.7)	1 (1)	
PCA	0 (0)	0 (0)	
Tandem lesion	20 (20.8)	13 (13.7)	0.191
**TOAST (%)**			0.753
Cardioembolism	61 (54.5)	54 (56.3)	
Large- artery atherosclerosis	28 (25)	20 (20.8)	
Other determined etiology	7 (6.3)	3 (2.1)	
Undetermined	16 (14.3)	16 (16.7)	
Collaterals (IQR)	3 (1-4)	3 (2-4)	0.217
Symptom to door (SD)	80.4 (58.8)	90.6 (62.9)	0.228
Door to imaging (SD)	33.9 (52.4)	23.9 (19.5)	0.078
Symptom to groin puncture (SD)	241.1 (221.6)	262.0 (163.0)	0.453
Door to groin puncture (SD)	160.1 (203.5)	174.0 (149.7)	0.591
Number of passes (IQR)	2 (1-4)	1 (1-3)	0.564
TICI 2b- 3 (%)	94 (85.5)	81 (84.4)	0.829
Stent	26 (23.2)	7 (7.3)	**0.02**
HT	18 (16)	21 (21.8)	0.250
HT PH2	5 (4.5)	6 (7.8)	0.756
Symptomatic ICH	4 (3.6)	2 (3.1)	0.593
NIHSS on admission (IQR)	15 (11-20)	14 (9-20)	0.231
NIHSS on discharge (IQR)	3 (1-9)	5 (2-8)	0.78
MRS baseline (IQR)	0 (0-0)	0 (0-0)	0.742
MRS discharge (IQR)	3 (1-5)	3 (2-5)	0.108
MRS 90 (IQR)	2 (1-4)	3 (2-5)	0.347
Mortality (%)	9 (8)	10 (10.4)	0.552
Outcome (favorable)	61 (58.1)	45 (47.9)	0.149

Values represent number of patients unless otherwise stated. Bold numbers signify statistically significant p values. SD = standard deviation, IQR = interquartile range, ICA = internal carotid artery, MCA = middle cerebral artery, ACA = anterior cerebral artery, PCA = posterior cerebral artery, HT = hemorrhagic transformation, PH = parenchymal hematoma. MRS = modified Rankin Scale.

Figure S1 – Study patient selection

Acute ischemic stroke patients

↓

Large vessel occlusion on CTA/MRA

↓

Eligible to receive intravenous thrombolysis

(Arrives within 4 hours from symptom onset, not treated with anticoagulation)

↓

Underwent Endovascular Thrombectomy

(ASPECTS≥6 for MCA territory stroke)
